# New Remedy for Tooth-Ache

**Published:** 1849

**Authors:** 


ARTICLE VIII.—*New Remedy for Tooth-Ache.*

Mr. James Beatson, of Airdrie, says: Gum copal when dissolved in chloroform, forms an excellent compound for stuffing the holes of decayed teeth. I have used it very frequently within the last two weeks, and the benefit which my patients have derived from it has been truly astonishing. The application is simple and easy. I clean out the hole, and moisten a little cotton with the solution; I introduce this into the decayed part, and in every instance, the relief has been almost instantaneous. The chloroform removes the pain, and the gum copal resists the action of the saliva, and as the application is so agreeable, those who may labor under this dreadful malady would do well to make trial of it.—*Med. Times* in *Jour. Dent. Sci.*

